# Association of a Brief Computerized Cognitive Assessment With Cholinergic Neurotransmission: Assessment Validation Study

**DOI:** 10.2196/68374

**Published:** 2025-07-07

**Authors:** Mouna Attarha, Ana De Figueiredo Pelegrino, Lydia Ouellet, Paule-Joanne Toussaint, Sarah-Jane Grant, Thomas Van Vleet, Etienne de Villers-Sidani

**Affiliations:** 1 Posit Science Corporation, 160 Pine St Suite 200, San Francisco, CA, 94111, United States, 1 267-930-4000; 2 Department of Neurology and Neurosurgery, McGill University, Montreal, QC, Canada

**Keywords:** cholinergic function, acetylcholine, FEOBV-PET, web-based cognitive assessment, brain health, cognitive status, assessment accessibility, anterior cingulate, computerized cognitive assessment, cholinergic neurotransmission, assessment validation study, self-administered, vesicular acetylcholine transporter, VAChT, positron emission tomography, PET, retrospective analysis, older person, neurotransmission, [18F]fluoroethoxybenzovesamicol

## Abstract

**Background:**

Computerized cognitive assessments are most often validated against standard neuropsychological measures with limited validation against biological indices of brain health.

**Objective:**

This study aimed to evaluate whether a self-administered computerized cognitive assessment is associated with cholinergic neurotransmission using the vesicular acetylcholine transporter ligand [18F]fluoroethoxybenzovesamicol (FEOBV) and positron emission tomography (PET).

**Methods:**

In a retrospective analysis, we report baseline data from the Improving Neurological Health in Aging via Neuroplasticity-Based Computerized Exercise (INHANCE) trial. This study provides normative data for healthy older adults aged 65 years and above. We evaluate the validity of the Double Decision cognitive assessment (from the BrainHQ assessment platform) by examining its association with tracer binding in the anterior cingulate cortex, as measured by FEOBV-PET. We also assess concurrent validity with neuropsychological performance using standardized measures of executive function and global cognition.

**Results:**

The intent-to-treat population from the INHANCE trial analyzed in this study included 92 healthy adults with a mean age of 71.9 (SD 4.86, range 65‐83) years, the majority of whom were female (61/92, 66%), with an average of 16.45 (SD 3.40, range 9‐27) years of education. The Double Decision assessment is associated with FEOBV binding in the anterior cingulate cortex, explaining 8% of the variance, and was associated with neuropsychological performance measures. The assessment was sensitive to age and was not influenced by education level or gender. Psychometric properties supported its usability and the assessment showed an average completion time of 3 (SD 1.12) minutes.

**Conclusions:**

We present the first brief, self-administered computerized cognitive assessment associated with cholinergic network health. This tool is scalable and accessible to individuals with an internet-connected device, offering a practical and cost-efficient approach to cognitive screening. The findings provide valuable insights into brain health, particularly for early detection of cognitive decline, and hold significant potential for broad applications across both clinical and nonclinical contexts.

## Introduction

Cholinergic function is recognized as a critical marker of overall brain health. Previous research has demonstrated that the cholinergic system regulates synaptic plasticity and is positively associated with key aspects of cognitive performance including sensory processing, attention, learning and memory, and executive function [1-6]. In contrast, cholinergic dysfunction is increasingly recognized as a precursor to cognitive decline, manifesting in subtle memory impairments before the onset of a clinically evident neurodegenerative disease [7-10].

Impaired cholinergic signaling is linked to broader neuropathological cascades central to conditions such as Alzheimer disease and related dementias [9,11,12]. Research indicates that it disrupts amyloid precursor protein metabolism, promoting amyloidogenic pathways that generate toxic amyloid-beta aggregates [11-18]. In addition, cholinergic decline is linked to heightened activity of glycogen synthase kinase-3, an enzyme involved in tau hyperphosphorylation and the subsequent formation of neurofibrillary tangles. These pathological processes fuel synaptic dysfunction, neuroinflammation, and neuronal loss, ultimately accelerating cognitive impairment. Conversely, activating cholinergic receptors demonstrates neuroprotective potential by steering amyloid precursor protein processing away from amyloidogenic pathways and mitigating tau pathology [11-18].

Given the cholinergic system’s central role in mediating and modulating cognitive performance [5,6,19-21] and its influence on amyloid-beta and tau-driven neurodegeneration [10], it has become a therapeutic target in clinical research [7,17,18,22,23]. Cholinesterase inhibitors are commonly prescribed to mitigate cognitive impairment [24]. By increasing acetylcholine availability in the synaptic cleft, these treatments aim to improve cholinergic signaling and preserve cognitive performance [24]. The therapeutic significance of acetylcholine highlights its fundamental role in maintaining cholinergic network health and underscores the need for early screening and detection of cholinergic dysfunction to develop timely and targeted interventions against cognitive decline.

Noninvasive methods for measuring cholinergic function in vivo in humans, however, remain a significant challenge. Most current approaches rely on neuroimaging techniques such as positron emission tomography (PET), which uses tracers to assess cholinergic receptor binding. While these methods provide valuable insights, they are costly, require specialized expertise, and expose participants to radiation, limiting their use in large-scale studies or routine clinical practice. To our knowledge, there are no validated, scalable, behavioral proxies of cholinergic function, particularly those using a brief, self-administered computerized cognitive assessment model.

In this study, we analyzed baseline data from the intent-to-treat (n=92) from the Improving Neurological Health in Aging via Neuroplasticity-Based Computerized Exercise (INHANCE) trial that was designed to evaluate the effect of cognitive training on cholinergic function in healthy older adults aged 65 years and above with a baseline Montreal Cognitive Assessment (MoCA) between 23 and 30. We assessed whether a cognitive assessment (Double Decision on the BrainHQ assessment platform) correlates with tracer binding levels in vivo within a prespecified region of interest (ROI), the anterior cingulate cortex [25], using PET imaging with the radiotracer [18F]fluoroethoxybenzovesamicol (FEOBV) [26]. Aging is linked to notable reductions in FEOBV binding, with an estimated decline of 2.5% per decade observed in the anterior cingulate cortex [27]. This region plays a critical role in supporting selective attention, learning and memory, and executive function [28-31]. We also examined whether the Double Decision assessment was associated with conventional measures of cognitive performance, specifically the MoCA [32] for global cognition and the National Institutes of Health Executive Abilities: Measures and Instruments for Neurobehavioral Evaluation and Research (NIH EXAMINER) battery for executive function [33]. We hypothesized that performance on Double Decision would be associated with cholinergic signaling and cognition.

## Methods

### Setting, Design, and Sample

Participants were recruited near McGill University, Canada, where the FEOBV radiotracer is synthesized and administered. Participants were community-dwelling, healthy older adults aged 65 years and above with a MoCA total score between 23 and 30 (both inclusive). The data reported herein reflect baseline assessment data of the intent-to-treat (N=92) from the INHANCE randomized clinical trial (refer to published protocol for full details [25]). The study enrolled from July 2021 to December 2023 with the final follow-up June 2024.

### Double Decision Assessment

**Figure 1.**
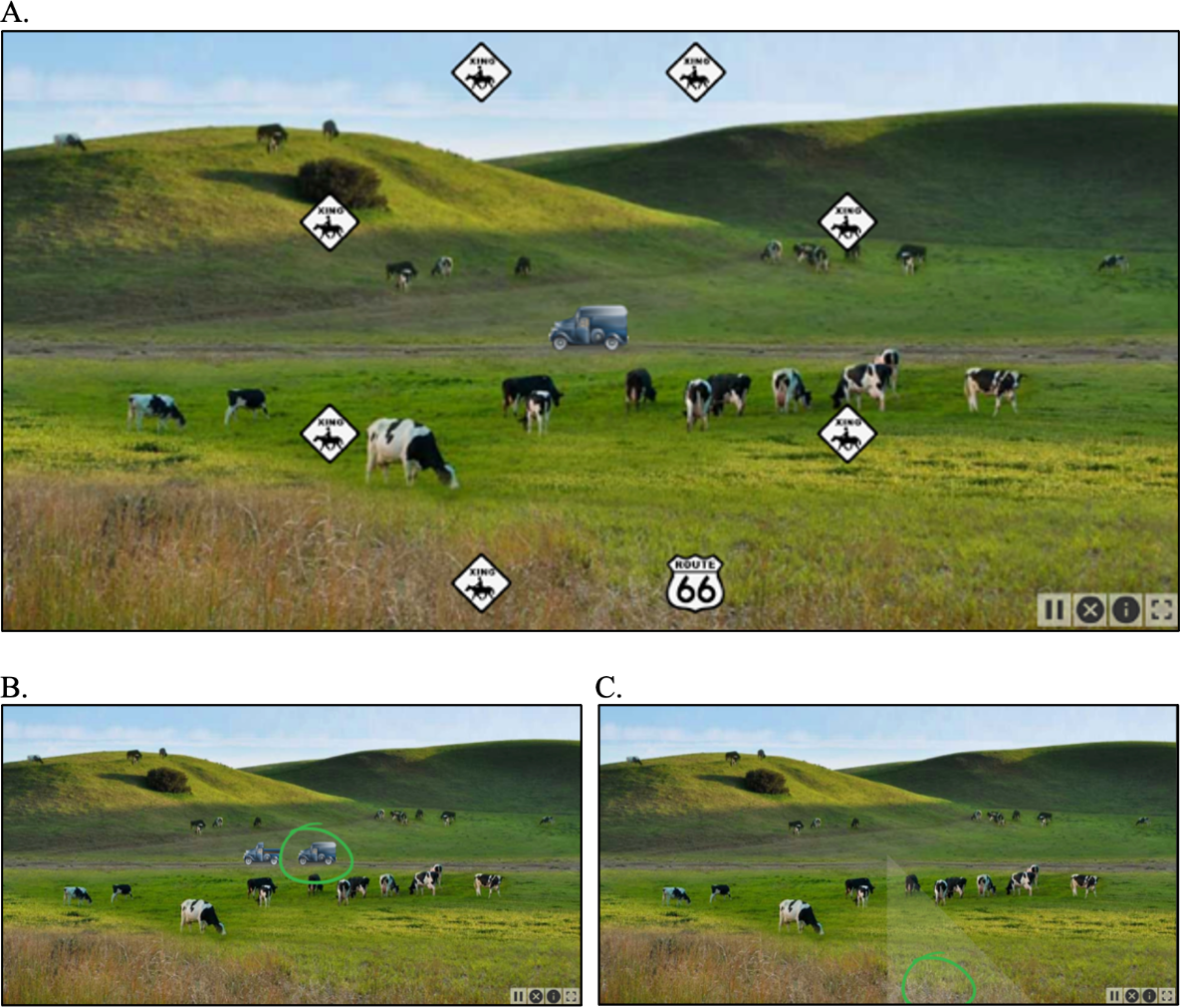
The Double Decision computerized cognitive assessment. (**A**) In this dual-task paradigm, participants simultaneously saw a vehicle at the center of the screen and signs in the periphery with a brief exposure duration. (**B**) Participants were asked to identify which vehicle (of 2) were presented and (**C**) pinpoint the location of the Route 66 sign (of 8 possible peripheral locations). Correct responses for the sample trial shown in (**A**) are highlighted in (**B**) and (**C**) for demonstration purposes.

The Double Decision assessment evaluates the visual speed of processing and selective attention [34]. This assessment is modeled upon the third subtest of the validated Useful Field of View (UFOV) assessment, which has established validity as a supervised in-clinic cognitive assessment and measure of driving safety [35-37]. Modifications include a modernized user interface with high-resolution color graphics and a guided flow to support self-administration (Figure 1). In this dual-task paradigm, participants discriminate a visual stimulus presented in the center of gaze while simultaneously locating a target in the peripheral visual field. The adaptive dimension is exposure duration (the length of time the stimuli remain visible on the screen). As the participant correctly responds to a trial, exposure duration decreases on subsequent trials, thereby making the task harder by requiring less time to process the visual display; conversely, as the participant responds incorrectly, exposure duration increases to make the task easier. Raw scores are defined as the exposure duration at approximately 80% criterion accuracy in milliseconds. The best possible raw score is 32 milliseconds, and the worst possible score is 3162 milliseconds.

### Ethical Considerations

The study was developed in accordance with the Declaration of Helsinki guidelines and was approved by the Western Institutional Review Board (IRB00000533) and Research Ethics Board of McGill University Health Centre (2020‐6474). The radioligand [18F]FEOBV was approved by Health Canada (Control # 252085). All participants provided written informed consent (Multimedia Appendix 1).

Study data were recorded into a secure, web-based electronic case report form at the study site through the Longitudinal Online Research and Imaging System. This system meets relevant privacy and security standards for electronic trial data entry and storage, as well as the Health Insurance Portability and Accountability Act and Personal Information Protection and Electronic Documents Act standards for confidentiality and privacy. Following consent, each participant was assigned a standardized Participant Identification Number composed of digits to identify the study and digits to identify the participant. All electronic case report form data entry were deidentified, using the Participant Identification Number and not the participant’s name.

Participants received CAD $40 (approximately US $30) for completing the baseline visit, which included the measures described below, on completion of the visit.

### Measures

Following informed consent, participants completed a structured clinical interview, the Double Decision assessment, 2 validated neuropsychological assessments to evaluate cognition (NIH EXAMINER and MoCA), and FEOBV-PET to evaluate cholinergic neurotransmission. Blinded staff members conducted the assessment administration and scoring.

A structured clinical interview (20 min, in person) collected demographic data (eg, age, gender, education).

Double Decision assessment (8 min, in person, computerized) evaluated visual speed of processing. The assessment was administered on a computer and scored automatically, with lower scores indicating better performance.

FEOBV-PET imaging (acquired 180 min post injection for 30 min, in person, scanner) evaluated cholinergic neurotransmission using mean standard uptake value ratios (SUVRs) for the primary ROI, the anterior cingulate, defined by the Hammers atlas [38]. Details are provided in the Imaging Acquisition and Processing subsubsection below.

NIH EXAMINER (20 min, in person, computerized) assessed executive function using a standard battery shown to have good reliability and validity. Performance was measured using the executive composite score [39] generated by the assessment program, with higher scores indicating better performance.

MoCA (10 min, in person, pen and paper) assessed global cognition. The MoCA includes visuospatial or executive functioning, naming, memory, attention, language, abstraction, delayed recall, and orientation to time and place for a total of 30 points, with higher scores indicating better performance.

### Imaging Acquisition and Processing

All participants underwent a structural T1-weighted magnetic resonance imaging (MRI) scan (3T Siemens Prisma) using the 3D magnetization-prepared rapid gradient echo sequence, followed by a [18F]FEOBV-PET scan using the Siemens High-Resolution Research Tomograph at the McConnell Brain Imaging Centre of the Montreal Neurological Institute-Hospital. The structural MRI was acquired during the imaging portion of the baseline visit to coregister the PET data [25].

Concurrent T1-weighted magnetization-prepared rapid gradient echo images were acquired with the related acquisition parameters being echo time (2.98 ms), repetition time (2300 ms), inversion time (900 s), flip angle (9°), isotropic resolution (1 mm), and image dimensions (192 mm×256 mm×256 mm).

Participants were positioned lying on their back for the PET imaging session and received a slow bolus intravenous injection of [18F]FEOBV with radioactivity doses ranging from 350 to 400 MBq [40], corresponding to 8.05 mSv-9.2 mSv, via a fine needle-catheter inserted into an arm vein. PET data acquisition started 180 minutes after injection, for a duration of 30 minutes, divided into 6 frames of 5 minutes each.

A transmission scan of 5 minutes was conducted with a rotating point source of [137Cs] for the Siemens High-Resolution Research Tomograph PET images in order to perform attenuation correction. PET images were reconstructed using an ordinary Poisson-ordered subset expectation maximization algorithm (10 iterations, 16 subsets) with resolution recovery, correcting for scatter, randoms, attenuation, decay, and dead time. Motion correction was applied, and time-averaged PET data (6×5-min frames) were used to create a static image. The final reconstruction was performed on a 256×256×207 matrix (voxel size: 1.22 mm³, spatial resolution: 2.3 mm full-width at half-maximum) with no postreconstruction smoothing or zoom.

PET preprocessing was performed using SPM12 in MATLAB. MRIs were segmented (gray matter, white matter, and cerebrospinal fluid), bias-corrected, and spatially normalized to the Montreal Neurological Institute (MNI) 152 asymmetrical template (MNI152 ONLine 2009 cAsym template) using Diffeomorphic Anatomical Registration Through Exponentiated Lie Algebra, with identical transformations applied to PET images. PET images were aligned to each participant’s MRI, and Müller-Gärtner partial volume correction was applied using the PETPVE toolbox. A 6 mm full-width at half-maximum Gaussian smoothing kernel was used to reduce noise.

SUVRs were used to quantify FEOBV binding. SUVRs were computed for spatially normalized PET images using a white matter mask as the reference region [41,42]. The Hammers atlas (MNI space) [38] was used to extract the SUVR in the target region (anterior cingulate cortex). A binary white matter mask was created and then eroded to reduce contamination from neighboring regions. The final eroded white matter mask was applied to the PET images to calculate mean tracer uptake for SUVR calculations.

### Analyses

#### Overview

Recruitment spanned from July 2021 to December 2023. The final follow-up visit was on June 7, 2024, and the study team was unblinded on June 14, 2024, after database lock. For all analyses, a *P*<.05 determined statistical significance with *P*<.10 reported as trending per the published statistical analysis plan [25].

#### Demographic Characteristics

Participants reported their age, gender, and educational level and completed the NIH EXAMINER and MoCA. Arithmetic means, SDs, and ranges are provided for continuous variables (age, education, NIH EXAMINER, and MoCA). For gender, we tallied the number of participants who selected a response option to each question and divided it by the total number of participants (N=92). We included both raw numbers and percentages for gender.

#### Assessment Characteristics

To evaluate usability, we reported general descriptive statistics (arithmetic mean and SD), distribution characteristics (skew and kurtosis), and psychometric properties, including the performance histogram of the proportion of participants achieving each assessment score, the mean number and SD of minutes spent in the assessment, and the percentage of participants obtaining the numerically lowest or highest assessment score to indicate the frequency of ceiling and floor effects.

#### Associations Between Demographics, FEOBV-PET, and Neuropsychological Tests

To establish associations between performance on the Double Decision assessment with demographic variables, we used the Spearman ρ for age and education, and the Wilcoxon rank sum for gender.

We used linear regression and Pearson *r* to establish the relationship between performance and FEOBV-PET SUVR within the anterior cingulate. PET image processing details are provided in the published protocol [25]. To establish the association between performance and standard neuropsychological measures, we used Pearson *r* for NIH EXAMINER and Spearman ρ for the MoCA.

## Results

### Demographic Characteristics

Participants (N=92) had a mean age of 71.90 (SD 4.86, range: 65‐83) years. Mean years of education was 16.45 (SD 3.40, range: 9‐27) years. A total of 61 participants of 92 identified as female (66%) and 31 participants of 92 identified as male (34%). The baseline NIH EXAMINER executive composite mean was 0.43 (SD 0.57, range: −1.50 to 1.52) and the baseline MoCA total score mean was 26.17 (SD 1.84, range: 23‐30) (Table 1). A full characterization of the intent-to-treat is presented by Pelegrino et al [43].

**Table 1.** Baseline characteristics of the intent-to-treat population (N=92).

Characteristic	Participant
Age (years), mean (SD)	71.9 (4.9)
Education (years), mean (SD)	16 (3.4)
Gender, n (%)	
Women	61 (66)
Men	31 (34)
Race, n (%)	
White	88 (96)
Asian	2 (2)
Black or African American	1 (1)
American Indian or Alaska Native	0 (0)
Native Hawaiian or other Pacific Islander	0 (0)
More than 1 race	0 (0)
Unknown or not reported	1 (1)
Ethnicity, n (%)	
Not Hispanic or Latino	91 (99)
Hispanic or Latino	0 (0)
Unknown or not reported	1 (1)
Double Decision (ms), raw score (SD)	1476.70 (813.06)
Anterior Cingulate, mean standard uptake value ratio (SD)	1.90 (0.19)
MoCA^a^, total score (SD)	26.2 (1.8)
NIH EXAMINER^b^, executive composite score (SD)	0.43 (0.57)

a MoCA: Montreal Cognitive Assessment.

b NIH EXAMINER: National Institutes of Health Executive Abilities: Measures and Instruments for Neurobehavioral Evaluation and Research.

### Assessment Characteristics

**Figure 2.**
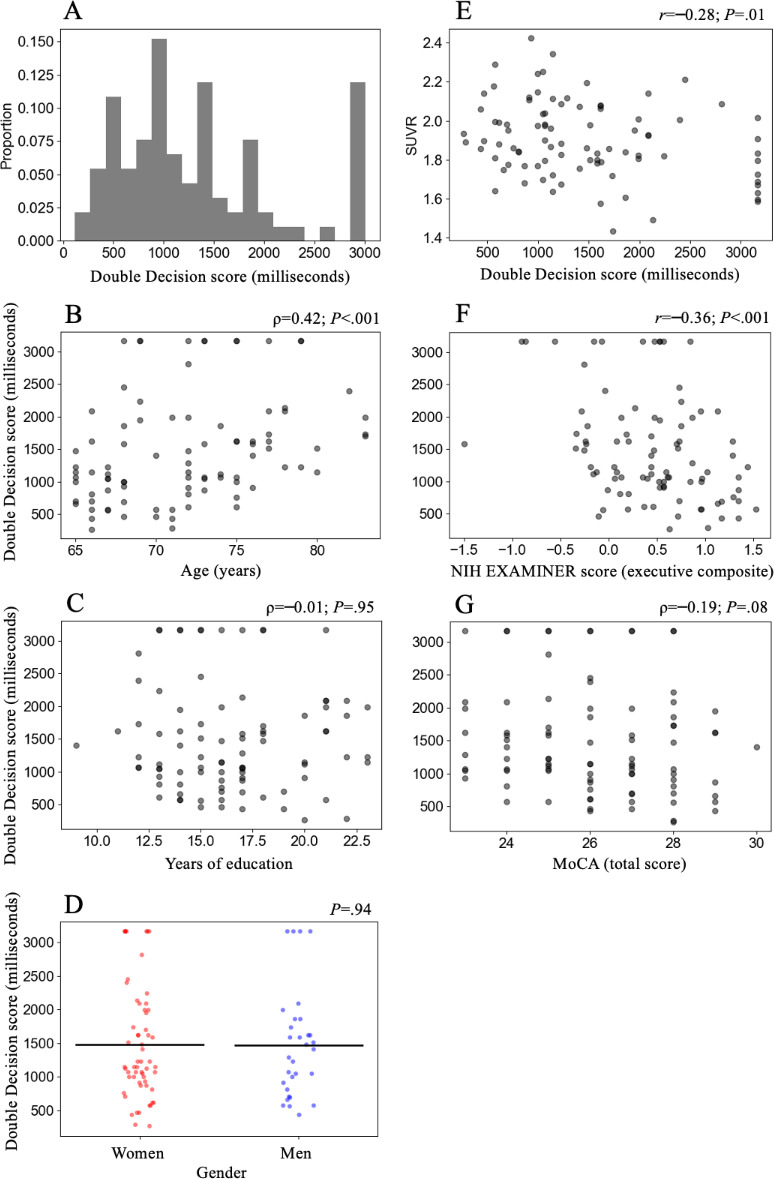
Outcome measures. (**A**) Performance histogram of Double Decision scores across participants (N=92). Association of Double Decision with demographic characteristics including (**A**) age, (**B**) years of education, and (**C**) gender. (**E**) Association of Double Decision with [18F]fluoroethoxybenzovesamicol–positron emission tomography standard uptake value ratios within the anterior cingulate cortex. (**F**) Association of Double Decision with the NIH EXAMINER executive composite, a standard validated measure of executive function. (**G**) Association of Double Decision with the MoCA, a standard validated measure of global cognition. MoCA: Montreal Cognitive Assessment; NIH EXAMINER: National Institutes of Health Executive Abilities: Measures and Instruments for Neurobehavioral Evaluation and Research; SUVR: standard uptake value ratio.

The performance distribution of Double Decision assessment scores is presented in Figure 2A. The mean score was 1476.70 (SD 813.06) milliseconds, with a skew of 0.84 and a kurtosis of −0.17. The minimum (best) score achieved among the 92 participants was 269 milliseconds (1.09% of participants), and the maximum (worst) score achieved was 3162 milliseconds (11.96% of participants). Participants took a mean of 3.17 (SD 1.12) minutes to complete the assessment.

### Associations Between Demographics, FEOBV-PET, and Neuropsychological Tests

There was a significant association of Double Decision with age (*r*=0.42; *P*<.001) and nonsignificant associations with education (ρ=−0.01; *P*=.95) and gender (*P*=.94) (Figure 2B-2D).

There was a significant negative association between Double Decision and FEOBV-PET SUVR (*r*=−0.28; *P*=.007) within the anterior cingulate cortex (Figure 2E). Linear regression showed that scores on Double Decision correlated with tracer binding (SUVR) using the following equation (*F*_1,87_=7.54; *P*=.007) and explained 8% of the variation in SUVR (*R*^2^=0.08).


(Double Decision score in ms)∗(−0.0000685)+2.0039=SUVR

There was a significant negative association between Double Decision and the NIH EXAMINER executive composite score (*r*=−0.36, *P*<.001) and a nonsignificant negative association between Double Decision and the MoCA total score (ρ=−0.19; *P*=.08) (Figure 2F-2G).

## Discussion

### Principal Findings

Double Decision is the first brief, self-administrable computerized cognitive assessment [44] validated against cholinergic binding in the anterior cingulate cortex using FEOBV-PET. Performance on Double Decision was negatively associated with SUVR, indicating that better (ie, lower) scores were correlated with higher cholinergic binding levels. This finding aligns with previous research showing a significant relationship between higher FEOBV-PET SUVR and cognitive performance, as measured by staff-administered validated computerized tools like the NIH EXAMINER [43], as well as traditional pen-and-paper measures [45,46].

Double Decision was further validated against standardized neuropsychological measures, showing a significant negative association with the NIH EXAMINER and a trending negative association with the MoCA. These results indicate that better (faster) performance (ie, lower scores) on Double Decision correlated with higher scores in executive function and global cognition. The assessment was brief, taking an average of 3 (SD 1.12) minutes to complete, and demonstrated good usability with reasonable descriptive and psychometric properties. It was sensitive to age within the narrow age band of 65‐83 years and was not influenced by demographic factors such as years of education or gender. The results support the adoption of this scalable form of biomarker-informed cognitive assessment available to individuals with an internet-connected device.

The Double Decision assessment is a gamified version of the third subtest from the well-known UFOV assessment. Meta-analyses have shown significant associations between UFOV and standard neuropsychological measures of general cognition, processing speed, attention, memory, and executive function [37]. UFOV has also been validated as a measure of at-fault motor vehicle crash risk [35,36]. The results of this study suggest that Double Decision and UFOV may assess biological markers of brain health that support a wide range of higher-order cognitive functions and real-world abilities.

### Significance and Implementation

This study represents an important step toward validating a noninvasive assessment of cholinergic function in humans. This brief, self-administrable assessment tool offers a scalable approach to cognitive screening, requiring only an internet-connected device. Broad accessibility makes Double Decision valuable for widespread use in community-based health initiatives, primary care settings, and clinical trials. It may be particularly beneficial for individuals residing in remote or underserved areas where access to traditional neuroimaging or comprehensive neuropsychological assessment may be limited, impractical, or cost-prohibitive.

The documented involvement of cholinergic dysfunction in neurodegenerative diseases [11], combined with the widespread use of cholinesterase inhibitors as a treatment for mild cognitive impairment, Alzheimer disease, and related dementias [24], highlights the essential role of acetylcholine as a therapeutic target to preserve the neurochemical integrity of the brain. The ability to digitally assess disruptions in cholinergic network health therefore represents an unmet need in the early detection and management of brain health and cognitive decline. This assessment could serve as an initial community-based screening tool for individuals at risk of cognitive decline, including adults experiencing subtle memory lapses, individuals with a family history of dementia, or those with other known risk factors such as cardiovascular disease or metabolic disorders. It may be used to monitor cognitive trajectories over time, identifying individuals who may benefit from further neuropsychological evaluation or early pharmacological or lifestyle interventions aimed at preserving cognition. In large-scale epidemiological studies, such a tool may facilitate the identification of population-level trends in cognitive health, enabling researchers to assess how various factors, such as genetics, lifestyle, or environmental exposures, contribute to cognitive aging. In clinical trials of cognitive impairment, it may serve as a measure to evaluate the efficacy of interventions aimed and altering cholinergic function.

By bridging a gap between neuroimaging biomarkers and widely deployable cognitive assessments, this study contributes to the field of digital biomarkers, offering a way to integrate remote cognitive screening into routine care. Ultimately, this approach may improve cognitive health monitoring, allowing for earlier identification of individuals at risk for cognitive decline and supporting the development of targeted intervention strategies to improve long-term brain health outcomes.

### Limitations

The study population had limited demographic variability. A total of 88 of 92 (96%) participants of the intent-to-treat identified as White; therefore, the results may not be generalizable to racial and ethnic minority groups [43]. Due to the study’s inclusion criteria, the age band (65 y and older) and the baseline MoCA total score (23-30) were range-limited.

### Strengths

This study represents the largest FEOBV-PET investigation to date, employing a rigorous design with stringent inclusion criteria and guided by a prespecified statistical analysis plan focused on a predefined ROI.

### Conclusion

Double Decision is the first brief and usable computerized cognitive assessment associated with cholinergic network health. This tool is scalable and accessible to any individual with an internet-connected device, offering a practical and cost-efficient approach to cognitive screening. The findings offer important insights into brain health, and may support the early detection of cognitive decline by serving as a behavioral proxy of forebrain cholinergic function. Future research should explore whether individuals with low performance on this assessment are at a higher risk of developing neurological or neurodegenerative disorders.

## Supplementary material

10.2196/68374 Multimedia Appendix 1 Consent form.

## Abbreviations

FEOBV: fluoroethoxybenzovesamicol
INHANCE: Improving Neurological Health in Aging via Neuroplasticity-Based Computerized Exercise
MNI: Montreal Neurological Institute
MoCA: Montreal Cognitive Assessment
MRI: magnetic resonance imaging
NIH EXAMINER: National Institutes of Health Executive Abilities: Measures and Instruments for Neurobehavioral Evaluation and Research
PET: positron emission tomography
ROI: region of interest
SUVR: standard uptake value ratio
UFOV: Useful Field of View

## References

[1] Picciotto MR, Higley MJ, Mineur YS (2012). Acetylcholine as a neuromodulator: cholinergic signaling shapes nervous system function and behavior. Neuron.

[2] Micheau J, Marighetto A (2011). Acetylcholine and memory: a long, complex and chaotic but still living relationship. Behav Brain Res.

[3] Himmelheber AM, Sarter M, Bruno JP (2000). Increases in cortical acetylcholine release during sustained attention performance in rats. Brain Res Cogn Brain Res.

[4] Parikh V, Bangasser DA (2020). Cholinergic signaling dynamics and cognitive control of attention. Curr Top Behav Neurosci.

[5] Logue SF, Gould TJ (2014). The neural and genetic basis of executive function: attention, cognitive flexibility, and response inhibition. Pharmacol Biochem Behav.

[6] Kilgard MP, Merzenich MM (1998). Cortical map reorganization enabled by nucleus basalis activity. Science.

[7] Hampel H, Mesulam MM, Cuello AC (2018). The cholinergic system in the pathophysiology and treatment of Alzheimer’s disease. Brain (Bacau).

[8] Muir JL (1997). Acetylcholine, aging, and Alzheimer’s disease. Pharmacol Biochem Behav.

[9] Singh S, Agrawal N, Goyal A (2024). Role of Alpha-7-Nicotinic Acetylcholine Receptor in Alzheimer’s Disease. CNSNDDT.

[10] Hampel H, Hardy J, Blennow K (2021). The amyloid-β pathway in Alzheimer’s disease. Mol Psychiatry.

[11] Mesulam M (2004). The cholinergic lesion of Alzheimer’s disease: pivotal factor or side show?. Learn Mem.

[12] Schliebs R, Arendt T (2006). The significance of the cholinergic system in the brain during aging and in Alzheimer’s disease. J Neural Transm (Vienna).

[13] Schliebs R, Arendt T (2011). The cholinergic system in aging and neuronal degeneration. Behav Brain Res.

[14] Orlando IF, Shine JM, Robbins TW, Rowe JB, O’Callaghan C (2023). Noradrenergic and cholinergic systems take centre stage in neuropsychiatric diseases of ageing. Neurosci Biobehav Rev.

[15] Berry AS, Harrison TM (2023). New perspectives on the basal forebrain cholinergic system in Alzheimer’s disease. Neurosci Biobehav Rev.

[16] Chaves-Coira I, García-Magro N, Zegarra-Valdivia J, Torres-Alemán I, Núñez Á (2023). Cognitive deficits in aging related to changes in basal forebrain neuronal activity. Cells.

[17] Shanbhag NM, Padmanabhan JL, Zhang Z (2025). An acetylcholine M1 receptor-positive allosteric modulator (TAK-071) in Parkinson disease with cognitive impairment: a phase 2 randomized clinical trial. JAMA Neurol.

[18] Anand P, Singh B (2013). A review on cholinesterase inhibitors for Alzheimer’s disease. Arch Pharm Res.

[19] Kilgard MP, Merzenich MM (1998). Plasticity of temporal information processing in the primary auditory cortex. Nat Neurosci.

[20] Kunnath AJ, Gifford RH, Wallace MT (2023). Cholinergic modulation of sensory perception and plasticity. Neuroscience & Biobehavioral Reviews.

[21] Hasselmo ME, Sarter M (2011). Modes and models of forebrain cholinergic neuromodulation of cognition. Neuropsychopharmacology.

[22] Kaul I, Sawchak S, Correll CU (2024). Efficacy and safety of the muscarinic receptor agonist KarXT (xanomeline-trospium) in schizophrenia (EMERGENT-2) in the USA: results from a randomised, double-blind, placebo-controlled, flexible-dose phase 3 trial. Lancet.

[23] Brannan SK, Sawchak S, Miller AC, Lieberman JA, Paul SM, Breier A (2021). Muscarinic cholinergic receptor agonist and peripheral antagonist for schizophrenia. N Engl J Med.

[24] Rockwood K (2004). Size of the treatment effect on cognition of cholinesterase inhibition in Alzheimer’s disease. J Neurol Neurosurg Psychiatry.

[25] Attarha M, de Figueiredo Pelegrino AC, Toussaint PJ, Grant SJ, Van Vleet T, de Villers-Sidani E (2024). Improving neurological health in aging via neuroplasticity-based computerized exercise: protocol for a randomized controlled trial. JMIR Res Protoc.

[26] Van Vleet TM, de Villers-Sidani E, Cote J, Merzenich M, Rosa-Neto P, Kang MS (2018). P2‐005: neuroplasticity‐based visual attention training and the expression of acetylcholine in healthy older adults. Alzheimer’s & Dementia.

[27] Albin RL, Bohnen NI, Muller M (2018). Regional vesicular acetylcholine transporter distribution in human brain: A [^18^ F]fluoroethoxybenzovesamicol positron emission tomography study. J Comp Neurol.

[28] Martinelli P, Sperduti M, Devauchelle AD (2013). Age-related changes in the functional network underlying specific and general autobiographical memory retrieval: a pivotal role for the anterior cingulate cortex. PLOS ONE.

[29] Pardo JV, Lee JT, Sheikh SA (2007). Where the brain grows old: decline in anterior cingulate and medial prefrontal function with normal aging. Neuroimage.

[30] Pardo JV, Nyabwari SM, Lee JT, Alzheimer’s Disease Neuroimaging Initiative (2020). Aging-related hypometabolism in the anterior cingulate cortex of cognitively intact, amyloid-negative seniors at rest mediates the relationship between age and executive function but not memory. Cereb Cortex Commun.

[31] Pezzoli S, Giorgio J, Martersteck A, Dobyns L, Harrison TM, Jagust WJ (2024). Successful cognitive aging is associated with thicker anterior cingulate cortex and lower tau deposition compared to typical aging. Alzheimers Dement.

[32] Nasreddine ZS, Phillips NA, Bédirian V (2005). The Montreal Cognitive Assessment, MoCA: a brief screening tool for mild cognitive impairment. J Am Geriatr Soc.

[33] Kramer JH, Mungas D, Possin KL (2014). NIH EXAMINER: conceptualization and development of an executive function battery. J Int Neuropsychol Soc.

[34] Jobe JB, Smith DM, Ball K (2001). ACTIVE: a cognitive intervention trial to promote independence in older adults. Control Clin Trials.

[35] Ball KK, Roenker DL, Wadley VG (2006). Can high-risk older drivers be identified through performance-based measures in a Department of Motor Vehicles setting?. J Am Geriatr Soc.

[36] Owsley C, Ball K, McGwin G (1998). Visual processing impairment and risk of motor vehicle crash among older adults. JAMA.

[37] Woutersen K, Guadron L, van den Berg AV, Boonstra FN, Theelen T, Goossens J (2017). A meta-analysis of perceptual and cognitive functions involved in useful-field-of-view test performance. J Vis.

[38] Hammers A, Allom R, Koepp MJ (2003). Three-dimensional maximum probability atlas of the human brain, with particular reference to the temporal lobe. Hum Brain Mapp.

[39] Possin KL, LaMarre AK, Wood KA, Mungas DM, Kramer JH (2014). Ecological validity and neuroanatomical correlates of the NIH EXAMINER executive composite score. J Int Neuropsychol Soc.

[40] Petrou M, Frey KA, Kilbourn MR (2014). In vivo imaging of human cholinergic nerve terminals with (-)-5-(18)F-fluoroethoxybenzovesamicol: biodistribution, dosimetry, and tracer kinetic analyses. J Nucl Med.

[41] Nejad-Davarani S, Koeppe RA, Albin RL, Frey KA, Müller M, Bohnen NI (2019). Quantification of brain cholinergic denervation in dementia with Lewy bodies using PET imaging with [^18^F]-FEOBV. Mol Psychiatry.

[42] Aghourian M, Legault-Denis C, Soucy JP (2017). Quantification of brain cholinergic denervation in Alzheimer’s disease using PET imaging with [^18^F]-FEOBV. Mol Psychiatry.

[43] Pelegrino A de F, Attarha M, Toussaint PJ (2025). Cholinergic neurotransmission in the anterior cingulate cortex is associated with cognitive performance in healthy older adults: baseline characteristics of the Improving Neurological Health in Aging via Neuroplasticity-based Computerized Exercise (INHANCE) trial. Neuroimage Rep.

[44] Attarha M, Mahncke H, Merzenich M (2024). The real-world usability, feasibility, and performance distributions of deploying a digital toolbox of computerized assessments to remotely evaluate brain health: development and usability study. JMIR Form Res.

[45] Xia Y, Eeles E, Fripp J (2022). Reduced cortical cholinergic innervation measured using [^18^F]-FEOBV PET imaging correlates with cognitive decline in mild cognitive impairment. Neuroimage Clin.

[46] van der Zee S, Müller M, Kanel P, van Laar T, Bohnen NI (2021). Cholinergic denervation patterns across cognitive domains in Parkinson’s disease. Mov Disord.

