# Supplementary material for: Association of a Brief Computerized Cognitive Assessment With Cholinergic Neurotransmission: Assessment Validation Study
Source: JMIR Form Res. 2025 Jul 7;9:e68374. doi: 10.2196/68374 (PMC12257569; doi:10.2196/68374)
Supplement: Multimedia Appendix 1 [file formative-v9-e68374-s001.pdf]

INFORMATION AND CONSENT FORM

**Research Study Title:**	Improving Neurological Health in Aging via Neuroplasticity-based Computerized Exercise (INHANCE)
**Protocol number:**	PSC-0903-19
**Researcher responsible for the research study:**	Etienne de Villers-Sidani, MD, FRCP(C)
**Co-Investigator(s)/sites:**	Jean-Paul Soucy, MD, MSc;<br>Thomas Van Vleet, Ph.D.<br>Mouna Attarha, Ph.D.
**Sponsor:**	Posit Science Corporation (PSC)<br>funded exclusively by the National Institute of Aging

INTRODUCTION

We are inviting you to take part in this research study because you are a person aged 65 years or older in generally good health, and we think you may be eligible to partake in our study to evaluate the effects of cognitive training programs (computerized programs) on the brain.

However, before you accept to take part in this study and sign this information and consent form, please take the time to read, understand and carefully examine the following information. You may also want to discuss this study with your family doctor, a family member or a close friend.

We invite you to speak to the researcher responsible for this study ("the researcher") or to other members of the research team and ask them any questions you may have about this study. Please also ask a member of the research team about any parts of this consent form you do not understand.

BACKGROUND

Americans and Canadians are living progressively longer lifespans; however, the onset of dementia does

not occur later just because people are living longer. Certain non-drug therapies have been shown to maintain brain health during older age. Most notably, forms of progressive computerized brain training (computer programs) that target specific areas and processes in the brain that affect one's focus and alertness have helped maintain brain health.

PURPOSE OF THE RESEARCH STUDY

The purpose of this study is to validate and evaluate how well two computerized cognitive training programs work on improving neurological and neuropsychological health in older adults.

We will look at whether the cognitive training has had any impact or change on the systems in your brain that deal with alertness and focus by taking PET images and MRI scans of your brain before and after your training period (explained in further detail later).

For this research study, we will recruit approximately 108 healthy participants, to ensure the successful completion of 90 participants. Participants will be men and women, aged 65 years and older.

DESCRIPTION OF THE RESEARCH PROCEDURES

This research study will take place at Montreal Neurological Institute-Hospital.

I. Duration and number of visits

Your participation in this research study will last about 6 months and will include 4-5 visits to the clinic. During your participation, you will engage in assigned training exercises for 7 sessions per week over 10 weeks with each session taking approximately 30 minutes to complete.

II. Overview of study participation

There are 6 periods to this study:

1. Screening Visit (V0)
- You will undergo a few screening assessments to determine if you qualify to participate in the study.
- You will come into the clinic for 1 visit.
- This visit will last approximately 1 hour.

2. Baseline Visit (V1):
- You will undergo additional procedures (the cognitive assessment, the PET scan and MRI scan)
- This period will last up to 4 weeks.
- You will come into the clinic for 1 visit, which may be split into 2 sessions, if needed.
- This visit will last approximately 3 hours.

3. Program Orientation and Intervention period

Before you start your training, we will invite you in for a program orientation session so that study staff may familiarize you to the training program in person.

We will also provide you with detailed, written information on how to perform the training program over the next several weeks.

- You will be randomly selected (like a flip of a coin) to participate in one of two groups for the computerized training programs and begin your daily training assessments for the next 10 weeks.
- This period lasts 10 weeks.
- You will come into the clinic for 1 visit, the program orientation visit. This visit may also be performed remotely through a phone or video call that the study staff will set up for you. The study staff will then follow-up with you during weekly check-ins to answer any questions and troubleshoot any issues. These check-ins will be conducted via email.
- This visit will last 1 hour.

4. Post-Intervention Visit (V2):
- You will have finished your 10 weeks of program training and come into the clinic for post-intervention assessments.
- You will no longer have access to the intervention applications after this visit.
- This visit will last approximately 3 hours and may be split into 2 sessions, if needed.

5. No-Contact Period
- Following the Post-Intervention Visit, you will be entered into a follow-up period lasting 3 months with no further program use. The study team will not be in contact with you other than to confirm the follow-up visit.

6. Follow-Up Visit (End of Study, V3)
- After the three months with no contact, we will invite you in for a final visit where you will complete some a final cognitive assessment.
- This visit will take place 6 months after your initial enrollment.
- This visit will last approximately 1.5 hours.

COMPUTERIZED TRAINING PROGRAMS

This study employs two computerized interventions: speed and attention training and executive function training. You will be assigned to one of the following groups:

Group 1: will do the speed and attention computer program training.
Group 2: will do the executive function computer program training.

This study is randomized, which means that you will be assigned to one of the groups. You may not choose the group to which you will be assigned; this process is done randomly, like flipping a coin. One person out of two (50%) will do the speed and attention computer program training, and one person out of two (50%) will do the executive function computer program training.

You will be required to participate in the assigned training exercises each day for 7 sessions per week over 10 weeks with each session taking approximately 30 minutes to complete.

Both can be done on any computer with an internet connection, and therefore within the comfort of your own home. If required, we can lend you a laptop computer or electronic tablet. You will be given the

opportunity to ask any questions about the computer program to ensure your proper understanding of how to use the program.

We will ask and remind you ***not*** to discuss details related to their training program with study staff, colleagues, friends, or acquaintances.

The following are some details to consider regarding the use of the program:

Location of Use: you may use the program at you place of residence or in clinic. We will offer you a loaner mobile device (tablet) from which you can use the program. You can otherwise opt to train on your personal device.

Fatigue: We expect that some participants may not be able to complete a full session which takes approximately 30 minutes to complete in one sitting. To accommodate this issue, participants may choose to break the time into shorter segments, such as 15 min in the morning and 15 min later in the day. Participants can pause the sessions to take a break at any time and continue where they left off.

You may discuss this with the study staff, who will work out a schedule that is most suitable for you. If ever you need to reduce the number of sessions per week, the study staff will work with you to generate a training schedule that works for you.

III. Study Procedures

During your participation in this research study, you will participate in the following procedures:

**DESCRIPTION OF STUDY PROCEDURES**	
**Procedure**	**Description**
Medical history and demographics	We will collect your complete medical history, including any medications you have used or are currently using and any other therapies or procedures you have had. Throughout the study you will be asked to report if you think that anything changes with your health and if anything has changed such as any new medications that you may have taken.<br><br>We will also ask you about age, your race and ethnic background.
Questionnaires and assessments	We will assess your cognitive status using the Montreal Cognitive Assessment (MoCA).<br><br>We will ask you some questions regarding the state of your mental health with the Geriatric Depression Scale (GDS) – Short Form and the Columbia-Suicide Severity Rating Scale (C-SSRS).
Neuropsychological assessments	A member of the study team will perform a neuropsychological assessment to measure specific age-related cognitive decline.

PET Scan and FEOBV radiotracer	PET is a nuclear medicine scanning procedure that involves the administration of very small amounts (tracer dose) of a chemical that will allow us to view specific areas of your brain (the FEOBV tracer). When administered intravenously, this chemical circulates in the blood to reach its target where it will briefly stay, before it decays. **The FEOBV radiotracer being used in this study is experimental, which means it has not been approved by Health Canada for use in regular medical practice.** However, Health Canada does not object to its use in this study.<br><br>This is a chemical tracer that is labeled with an atom which allows for it to be detected on the PET camera and provide a clearly defined image of your specific areas of your brain.<br><br>During this process, the tracer will emit a very small amount of radioactivity that can be detected by a sophisticated PET camera. With the help of high-power computing, researchers are then able to study the distribution within brain of the chemical that has been administered. **No effect of the chemical can be detected in a given individual, since it is always administered in very small amounts (tracer dose).**<br><br>All PET imaging sessions scheduled as part of your participation will be supervised by a qualified nuclear medicine technician. A physician is available, if needed. On your arrival at the MNI PET unit, you will have to fill in routine questionnaires about your general physical condition. Then, a fine needle-catheter will be inserted into an arm vein. This catheter will be used for the administration of FEOBV.<br><br>You will then have to wait for approximately 180 minutes for the chemical to be appropriately distributed in your brain. During this time, you should remain at rest, but will be able to use the washroom and walk around if necessary.<br><br>You will then be asked to lie down on a bed that will be moved into a cylindrical opening for the scanning process, which lasts approximately 40 minutes.<br><br>The device is completely passive and has no electrical (or other) output that may be harmful.<br><br>The scans will be completed at the MNI PET Unit, or at the Concordia PERFORM Centre (7200 Sherbrooke St. W., Montreal, QC, Canada, H4B 1R2), depending on the availability of the facility. The study team will let you know where your scans will occur.

	If your scan is conducted at the PERFORM Centre, following the administration of FEOBV at the MNI, you will be transferred to the PERFORM Centre. The study team can organise transport (taxi), or you may drive yourself.
MRI	This is a test that uses a magnet and radio waves to produce a detailed scan (picture) of the brain and spine. An MRI scanner looks like a large doughnut-shaped magnet that often has a tunnel in the center. You will be asked to lie down on a table that slides into the tunnel. Your head will be supported by pillows. Please let the study staff know if you get uncomfortable or frightened when in small spaces.<br><br>Please let the study staff know if you have any implanted devices.<br><br>Inside the scanner you will hear a fan and feel air moving. You may also hear tapping or snapping noises as the scans are taken. You will be given earplugs or headphones with music to reduce the noise. It is very important to hold as still as possible while the scan is being done. You may be asked to hold your breath for short periods of time.<br><br>You will be provided with a call button so that you can communicate with the MRI team at any time and you will be able to talk to the team via an intercom between scans.<br><br>Your study staff will give you detailed instructions on how to prepare for your scans. You will need to remove all metal from your body and change into scrubs.<br><br>This scan may take up to 20 minutes.
Behavioral Assessment	To assess for acetylcholinergic function (how your muscles respond), we will measure your heart rate variability and/or pupillometry (the widening of your pupils) during train-to-task assessments or as needed.<br><br>The pupillometry acquisition will be carried out using Tobii Pro Glasses 2. These are glasses that you will have to wear; in doing so, they will monitor your eye movements.<br><br>The heart rate variability will be acquired using a wearable wrist band monitor.
Email communication with study staff	This study will rely on the use of email communication between study staff and research participants as part of their participation in the clinical trial.

	Study staff are expected to email participants about their upcoming appointments, provide weekly updates on program usage, or communicate other important study information, including the instructions for completing study activities remotely.<br><br>Participants may also ask questions of study staff using email.
Secure mobile app usage	We are aware that mobile app use can pose some confidentiality hazards to users and hence put in place several important safeguards to minimize potential risks.<br><br>Security of electronic data is ensured at the level of the server, the user, and the database.<br><br>We will provide you with de-identified log-in to access the training program. The program will not capture, collect, transmit or store personally identifiable data, except for dates that training exercises are completed. The computerized program also does not include geographic location data.

PARTICIPANT'S RESPONSIBILITIES

- Attend all study visits.
- Complete one training session each day, each session will take approximately 30 minutes to complete.
  - Taking a pause or breaking up the session throughout the day is permitted.
- Check your emails and respond to the coordinator 'check-in' messages.
- Follow the instructions of the study investigator and staff.
- Please do not discuss the details of the computerized training program with anyone.

BENEFITS ASSOCIATED WITH THE RESEARCH STUDY

There is no direct benefit to you for participating in this research. However, we hope that the study results will contribute to the advancement of scientific knowledge in the study field.

RISKS ASSOCIATED WITH THE RESEARCH STUDY

A possible risk associated with this study is a breach of confidentiality or use of your personal information by a third party. To limit this risk, we will take the steps to protect your confidentiality described in the Confidentiality section, below.

<u>Discomfort During Assessments and Training.</u>
Computerized assessments and training may be fatiguing or frustrating for some individuals. To minimize this potential discomfort, breaks are encouraged and scheduled within the session. You may discontinue testing sessions at any time if you fee to be under undue strain.

<u>*Lack of Assessment Feedback*</u>.
Participation in this study does not include feedback to participants on their individual assessment results, which may be frustrating.

<u>*Risks of Email Communication*</u>.
There are risks associated with email communication, and these risks increase when emails are sent without an encryption service. Risks of sending or receiving unencrypted emails include, but are not limited to:

- Others can intercept messages.
- If messages are sent or received on an employer-owned device, the employer may have the right to save and read the messages. The internet or cell-phone provider may also have the right to save and read email messages.
- A copy of the message may be saved on a device or computer system, even if it is deleted.
- If an email address is not typed correctly, it can be sent to the wrong person.
- Emails can spread computer viruses.
- Others may be able to access messages on devices that were lost, stolen, or thrown away.
- If a user changes emails without notifying study staff, they may miss communications.

<u>*Loss of Privacy*</u>
One of the risks to the participants are those that would follow a breach of confidentiality and the disclosure of clinical information. Participation in any research study, including this one, may involve a loss of privacy. Procedures designed to maintain data confidentiality include (1) formal protocol training sessions for all study staff members emphasizing the importance of confidentiality, (2) adherence to specific procedures developed to protect participants' confidentiality, and (3) formal mechanisms limiting access to information that can link data to individual participants.

<u>*PET scan*</u>
During the PET imaging sessions, participants may feel a stinging sensation at the time of the catheter insertion into the vein. As with any other type of injection, there is a minimal chance of infection on the site of injection. To limit this risk, sterile equipment will be used on a thoroughly disinfected skin area. Moreover, the prolonged immobility on the couch may also be a source of restlessness and discomfort for some participants. PET imaging involves the injection of specific agents (FEOBV) not normally present in the human body. Like any other chemical or pharmaceutical compound, these agents have a potential to produce undesirable or allergic reactions. However, such reactions have never been observed with the doses to be used in this study.

Given that FEOBV is a radioactive compound, this means that participants will be exposed to a small dose of radiation (measured in milliSieverts, or mSv), above what one individual is usually exposed to in daily life (natural radiation in the environment, cosmic rays, etc.), or for medical reasons (diagnostic X-rays, radiation therapy, etc.). Most of the radioactivity will be gone from the body after a few hours (by 20 hours, it will be essentially undetectable). The risk which is alluded to when discussing risk associated with radiation exposures of the level seen in PET scanning is that of developing a cancer at some point in the future, which would not have developed otherwise. As a general concept, it is known that radiation increases the risk of developing cancer over certain doses. However, because of the very small doses used for PET imaging, this has never been observed. The risk is therefore low. The FEOBV radiotracer being used in this study is experimental, which means it has not been approved by Health Canada for use in regular medical practice. However, Health Canada does not object to its use in this study. **Nationally**

accepted limits of radiation doses administered for research purposes have been defined at 50 mSv per year, so you need to inform the study team of all the scans you have undergone in the last 12 months, so as not to exceed the limit.

You are also required to mention your participation in this study to any investigator asking you to take part in a study involving radiation. The dose you are expected to receive for the 2 PET scans as part of the study is estimated at 11-15.4 mSv.

Risks specific to FEOBV:
No adverse reactions to FEOBV have been reported yet. However, there are some potential risks as listed below:

Risks of FEOBV for pregnant women, unborn children or to children of breastfeeding women are not known, therefore FEOBV should be avoided in pregnant and breastfeeding women. Consequently, pregnant and breastfeeding women may not participate in this study.

Given that FEOBV is a radioactive compound, traces of the small amount participants will be exposed to can appear in urine and fecal matter and risk contaminating of the surfaces it touches. For up to 12 hours after the administration of FEOBV, a toilet should be used instead of a urinal, and the toilet must be flushed several times after use.

MRI
During this test, you will be exposed to a strong magnetic field and radio waves. However, no long-term negative side effects have been observed for this type of exam.

An MRI scan can be a rather noisy procedure, and some people may feel uncomfortable while lying in the scanner. You will be given ear plugs to block out the noise.

You may briefly experience claustrophobia (a closed-in feeling) during the MRI procedure. Some people may find the combination of the noise and the feeling of being “closed in” uncomfortable. You will be in constant communication with the MRI technician, and you will be provided with a “call button” if you do not feel well and want the procedure to stop.

Please inform the study staff if you have one of the following contraindications:
- Pacemaker
- Aneurysm clip
- Heart/vascular clip
- Prosthetic valve
- Metal prosthesis
- Pregnancy or intend to become pregnant
- Metal fragments in body
- Transdermal patches (must be removed prior to exam, please bring an additional patch to apply after the scan)

INCONVENIENCES LINKED TO STUDY PROCEDURES

These are the only foreseeable inconveniences that may result from study participation:

- Time commitment required to complete the trainings daily.

VOLUNTARY PARTICIPATION AND THE RIGHT TO WITHDRAW

Your participation in this study is voluntary. Therefore, you may refuse to participate. You may also withdraw from the *ongoing* project at any time, without giving any reason, by informing a member of the study team. Your decision not to participate in the study, or to withdraw from it, will have no impact on the quality of care and services to which you are otherwise entitled. You will be informed in a timely manner if any information becomes available that may impact your willingness to continue participating in this study.

The researcher or the Research Ethics Board may put an end to your participation without your consent. This may happen if new findings or information indicate that participation is no longer in your interest, if you do not follow study instructions, or if there are administrative reasons to terminate the project.

If you withdraw or are withdrawn from the study, you may also request that the data already collected about you be removed from the study.

You may choose to stop using the computer program but continue to partake in the study and come in for a *Post-Intervention Visit (V2)* and *Follow-up Visit (V3).*

Any new findings that could influence your decision to stay in the research project will be shared with you as soon as possible.

CONFIDENTIALITY

During your participation in this study, the researcher and his/her team will collect and record information about you. They will only collect information necessary for the study.

The following information may be collected: information from your medical chart, including your identity, concerning your past and present state of health, your lifestyle, as well as the results of the tests, exams, and procedures that you will undergo during this research project. Your research file could also contain other information, such as your name, sex, age, date of birth, ethnic origin and other dates from data collected (such as dates for completion of assessments and training exercises).

All the information collected during the research project will remain confidential to the extent provided by law. You will only be identified by a code number. The key to the code linking your name to your study participant number will be kept by the researcher.

To ensure your safety, a confirmation of participation in clinical research form will be placed in your medical chart. As a result, any person or company to whom you give access to your medical chart will have access to this information.

The researcher may forward your coded data to the sponsor. However, once coded data is transferred outside of Canada, protections equivalent to those in Canada and Quebec will be enforced.

The study data will be stored for 25 years by the researcher responsible for the study.

The data may be published or shared during scientific meetings; however, precautions will be taken to ensure that it will not be possible to identify you.

For auditing purposes, the research study files which could include documents that may identify you may be examined by a person mandated by the study sponsor, the institution, or the Research Ethics Board and the NIH. All these individuals and organizations adhere to policies on confidentiality.

Data Archive

Two years after the completion of the trial, we will store all of the coded study data, including MRI and PET data, from all the participants in the study on a platform that has been vetted and approved by the institution. To protect your confidentiality, imaging data will be defaced, which means your face in the images will be masked.

Storing all the study information in a database for this trial will allow other academic researchers who were not affiliated with the trial to conduct further analyses on the research results. This will only happen two years after the study is over so that the objectives of the main study are all analyzed before other non-affiliated researchers have access to the data.

Sharing the data on an institutionally vetted database is open; this means any researcher can access the study data either freely or after registering with the platform. However, as mentioned in the Confidentiality section, any data that may be published or shared will have the precautions in place to ensure your identity is kept confidential.

INCIDENTAL FINDINGS

Material incidental findings are unexpected findings made in the course of the study that may have significant impacts on your current or future wellbeing or that of your family members. A material incidental finding concerning you in the course of this research will be communicated to you and to a health professional of your choice.

FUNDING OF THE RESEARCH PROJECT

The researcher and the institution have received funding from the National Institute of Aging to conduct this research project.

MARKETING POSSIBILITIES

The computerized training programs being studied are developed by Posit Science Corporation. The Principal Investigators at Posit Science Corporation may benefit from commercial activities that may later derive from the results of this research. However, you will not receive any financial benefits.

CONFLICT OF INTERESTS

The Principal Investigators are employees of and hold stock in Posit Science Corporation. The Principal Investigators at Posit Science Corporation may benefit from commercial activities that may later derive from the results of this research.

COMPENSATION

You will receive compensation for costs and inconveniences incurred during this research study. If you withdraw from the study, or are withdrawn before it is completed, you will receive compensation proportional to the number of visits you have completed.

The breakdown of reimbursement is as follows:

- Baseline Visit including PET imaging (V1): $40
- You will receive $13 for every 10 training session completed (maximum of $91 for completing all 70 sessions) during the intervention period.
- Post-intervention visit including PET imaging (V2): $40
- Follow-up visit (V3): $40

Participants who complete the study in its entirety will be reimbursed $211

Participants who complete the PET imaging at the Concordia PERFORM Centre will receive an additional 50$ for transportation reimbursement at these visits.

For participants who are loaned a tablet, compensation for all study activities completed through the end of the *Intervention Period* will occur after the participant returns the loaned tablet to the study staff at the *Post-Intervention Visit* (V2). Participants who withdraw from the study will be compensated for all study visits they have completed once the loaned tablet has been returned to the study staff.

SHOULD YOU SUFFER ANY HARM

Should you suffer harm of any kind following any procedure related to the research study, you will receive the appropriate care and services required by your state of health.

By agreeing to participate in this research project, you are not waiving any of your legal rights nor discharging the researcher, the sponsor or the institution, of their civil and professional responsibilities.

CONTACT INFORMATION

If you have questions or if you have a problem you think may be related to your participation in this research study, or if you would like to withdraw, you may communicate with the researcher or with someone on the research team at the following number: 514-398-2964

For any question concerning your rights as a research participant taking part in this study, or if you have comments, or wish to file a complaint, you may communicate with the Montreal Neurological Hospital Patient Ombudsman at the following phone number: (514) 934-1934, ext 48306.

OVERVIEW OF ETHICAL ASPECTS OF THE RESEARCH

The McGill University Health Centre Research Ethics Board reviewed this research and is responsible for its ongoing ethical oversight.

Research Study Title: Improving Neurological Health in Aging via Neuroplasticity-based Computerized Exercise (INHANCE)

SIGNATURES

Signature of the participant

I have reviewed the information and consent form. Both the research study and the information and consent form were explained to me. My questions were answered, and I was given sufficient time to make a decision. After reflection, I consent to participate in this research study in accordance with the conditions stated above.

I authorize the study team to have access to my medical record for the purposes of this study.

I authorize a member of the research study to contact me in the future to ask if I am interested in participating in other research.

Yes ☐ No ☐ If yes, please provide contact information: __________________________

Name of participant	Signature	Date

Signature of the person obtaining consent

I have explained the research study and the terms of this information and consent form to the research participant, and I answered all his/her questions.

Name of the person obtaining consent	Signature	Date
